# DJ-1 Oncogene as a Potential Diagnostic and Prognostic Biomarker for Head and Neck Cancer

**DOI:** 10.7759/cureus.36229

**Published:** 2023-03-16

**Authors:** Rey A De La Torre, Mourad Kerdjoudj, Hilal Arnouk

**Affiliations:** 1 Osteopathic Medicine, Midwestern University Arizona College of Osteopathic Medicine, Glendale, USA; 2 Medicine, Midwestern University Chicago College of Osteopathic Medicine, Downers Grove, USA; 3 Pathology, Midwestern University Chicago College of Osteopathic Medicine, Downers Grove, USA; 4 Pathology, Midwestern University Chicago College of Graduate Studies, Downers Grove, USA; 5 Molecular Pathology, Midwestern University Chicago Precision Medicine Program, Downers Grove, USA; 6 Pathology, Midwestern University Chicago College of Dental Medicine, Downers Grove, USA; 7 Pathology, Midwestern University Chicago College of Optometry, Downers Grove, USA

**Keywords:** oral cancer, oscc, oral squamous cell carcinoma, precision medicine oncology, computer-assisted image analysis, immunohistochemistry (ihc), prognostic biomarkers, diagnostic biomarkers, head and neck cancer, dj-1

## Abstract

Background

Current methods used to diagnose and prognosticate oropharyngeal cancer have contributed to unfavorable patient survival rates that have not significantly improved for the last several decades. Precision medicine oncology relies on molecular diagnostics and biomarkers to supplement existing methods of detecting and prognosticating cancers. This study evaluated the expression of DJ-1, an oncogene that is implicated in the pathogenesis of oral squamous cell carcinoma (OSCC), the most common type of head and neck cancer, to determine its utility as a diagnostic and prognostic biomarker.

Methodology

Immunohistochemistry (IHC) was performed on 13 normal oral mucosa tissue samples and 143 OSCC tissue samples of varying histopathological grades. Computer-assisted image analysis was performed using the Aperio ImageScope software from Leica Biosystems (Buffalo Grove, IL), which utilizes an algorithm of positive pixel counting for the quantification of immunoreactivity and the percentage of positive cell staining, generating a histo-score (H-score). The comparisons of the average H-scores of the different groups were made using a two-tailed T-test with P ≤ 0.05 set as the level of significance.

Results

The study found a significant increase in DJ-1 expression in oral squamous cell carcinoma tissue samples in comparison to the normal oral mucosa tissue samples. Additionally, the study documented a significant upregulation in DJ-1 expression in the OSCC tissue samples with high histopathological grades compared to the OSCC tissue samples with low histopathological grades.

Conclusions

DJ-1 expression patterns were able to reliably differentiate between oral squamous cell carcinoma and the normal counterpart tissues of the oral mucosa, thereby highlighting its role as a potential diagnostic biomarker. Moreover, DJ-1 expression significantly correlates with the OSCC histological grade, which serves as an indicator of the differentiation status and a predictor of the biological behavior of malignant neoplasms, adding to DJ-1's potential utility as a prognostic biomarker for this common type of head and neck cancer.

## Introduction

Oral squamous cell carcinoma (OSCC), which affects the mucosal layer of the oral cavity and pharynx, falls under the category of head and neck cancers. Cancers of the oral cavity make up more than the majority of head and neck cancers [1]. The major contributing risk factors identified for OSCC have been linked to alcohol consumption and tobacco use [2]. The relative five-year survival rate for oropharyngeal cancer is estimated to be 67.4% based on the Surveillance, Epidemiology, and End Results (SEER) program data [3]. However, an individual patient's relative survival can vary depending on the progression of cancer and whether it has spread beyond the site of origin. Specifically, patients tend to have a higher survival rate when the cancer is contained in situ compared to patients where cancer has spread and metastasized to lymph nodes or other areas of the body [4].

The histopathological grade of OSCC can be characterized into numerical grade based on the degree of differentiation and the resemblance to normal oral epithelium [5]. Thus, the histopathological grade can inform about the biological behavior of tumors, which tends to correlate inversely with the differentiation status. However, the assignment of a certain histopathological grade based on morphological features alone can be subjective and prone to inter-observer variability [6]. In order to enhance the predictive power of pathological evaluations, molecular biomarkers can be used to supplement the morphological examination of tumor tissues as prognostic indicators [7-9].

The protein deglycase DJ-1, also known as Parkinson's disease protein 7 (PARK-7), was identified as an oncogene that is upregulated in several types of cancer [10-12]. It has been shown that DJ-1 overexpression in glottic and supraglottic cancers can be linked to an overall poor prognosis, thereby underscoring its potential as a prognostic biomarker [13,14]. This study aims to examine the utility of DJ-1 as a prognosticator for oral squamous cell carcinoma, a malignant head and neck neoplasm that has not seen improvement in survival rates for decades due primarily to the lack of early detection.

## Materials and methods

Immunohistochemistry (IHC) staining

This study utilized paraffin-embedded de-identified tissue microarrays that were acquired from US Biomax and US Biolabs (Rockville, MD) and contained 13 normal oral mucosa tissue samples and 143 tissue samples of oral squamous cell carcinoma (OSCC) with varying histopathological grades (80 low-grade OSCC and 63 high-grade OSCC). The experimenters were blinded to information about the tissue slides throughout the steps of immunohistochemistry (IHC) staining and computer-assisted image analysis. The antigen retrieval step was performed using a steamer before blocking with bovine serum albumin (BSA) for one hour. The tissue samples were then labeled with anti-DJ-1 antibody (catalog number 11681-1-AP; Proteintech, Rosemont, IL) at 1:200 dilution followed by incubation with a corresponding secondary antibody conjugated to horseradish peroxidase (HRP) (catalog number A9169; Sigma-Aldrich, St. Louis, MO) at a 1:200 dilution. Finally, immunoreactivity was visualized using the chromogenic substrate, 3,3'-diaminobenzidine (DAB), followed by hematoxylin counterstaining and sealing with a coverslip for preservation.

Quantitative analysis of DJ-1 expression

High-resolution images of the IHC-stained tissue samples were taken using a Nikon A1R (Nikon Corporation, Tokyo, Japan) inverted microscope at 10× magnification and then analyzed using the Aperio ImageScope software (Leica Biosystems, Buffalo Grove, IL) to generate immunoreactivity score (histo-scores {H-scores}), with a pixel counting algorithm built in, to eliminate the subjectivity in assigning immunoreactivity scores that are typically associated with manual scoring methods. After the stroma and other connective tissues were excluded from the analysis, immunoreactivity was calculated based on the percentage of positive pixel count and pixel intensity to generate an H-score [15,16]. Mean H-scores were recorded and assigned to tissue samples, which were first grouped into normal or cancer tissue samples, based on their histopathological grade within the cancer tissue sample collection. Comparisons were made using a two-tailed Student's unpaired T-test with a comparison being considered statistically significant at p ≤ 0.05.

## Results

DJ-1 expression in oral squamous cell carcinoma versus the normal oral mucosa

**Figure 1:**
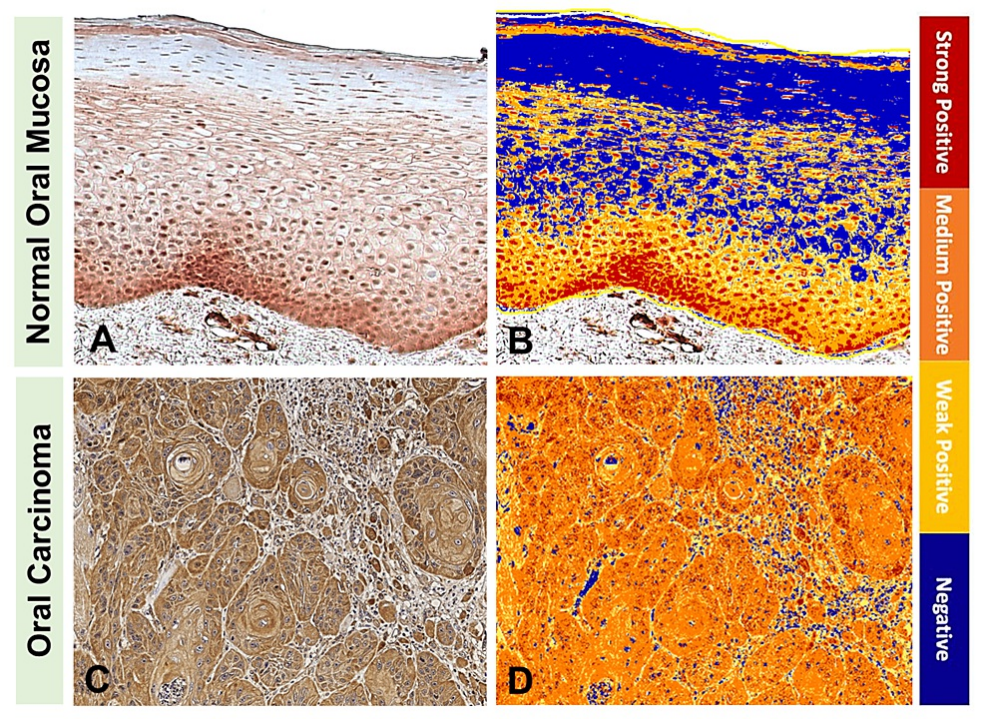
DJ-1 expression in the normal oral mucosa versus oral squamous cell carcinoma tissue samples. The upper panel shows a representative normal oral mucosa tissue sample: (A) immunohistochemistry for DJ-1 immunoreactivity and (B) ImageScope computer-assisted analysis. The lower panel shows a representative oral squamous cell carcinoma tissue sample: (C) immunohistochemistry for DJ-1 immunoreactivity and (D) ImageScope computer-assisted analysis.

Computer-assisted image analysis of DJ-1 immunoreactivity showed that DJ-1 consistently displayed a unique staining pattern in normal oral mucosa samples, where it stained intensely in the basal layers of the oral mucosa in comparison to other layers of keratinocytes of the same sample. On the other hand, OSCC samples displayed positive DJ-1 staining that was more diffused and lacks any discernable pattern (Figure 1).

**Figure 2:**
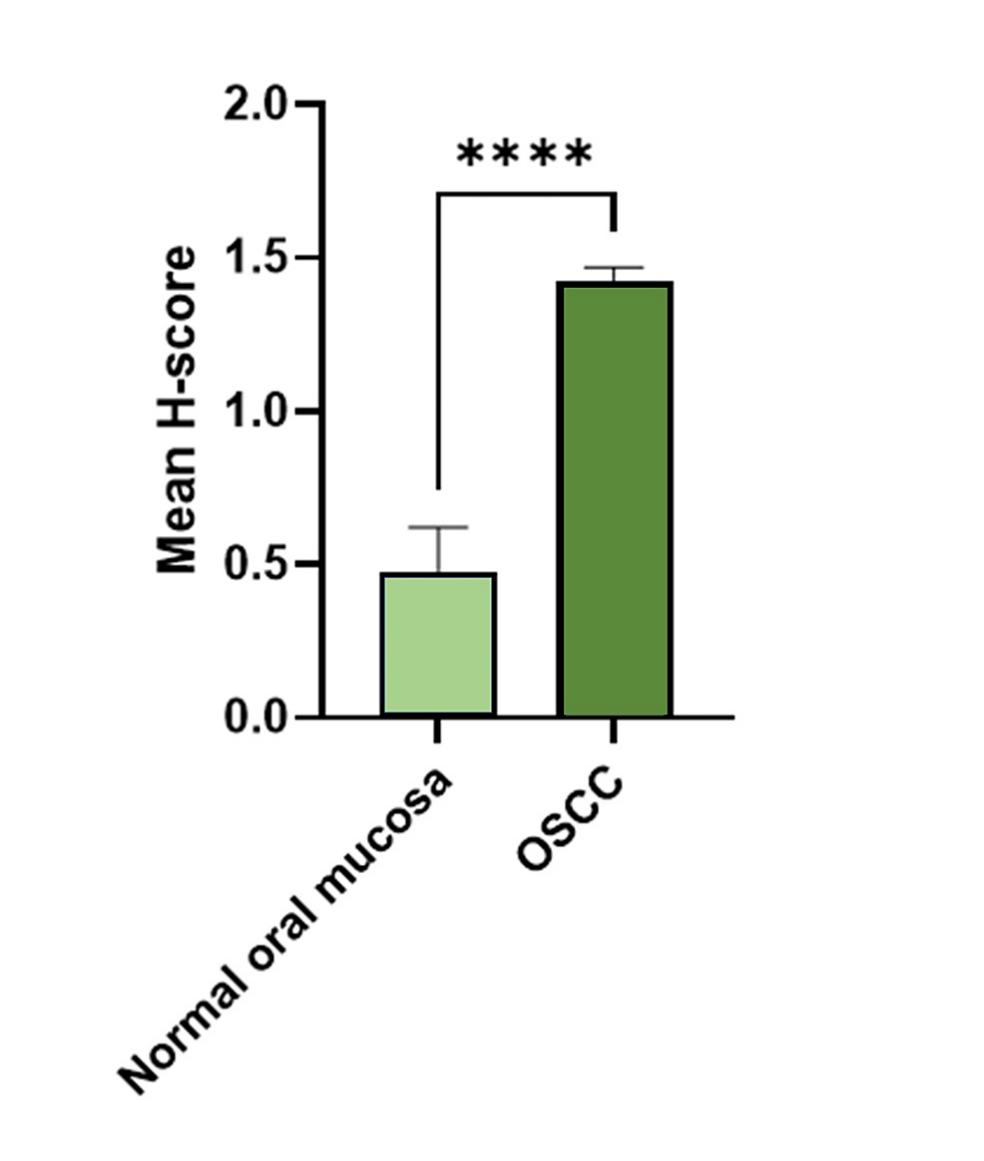
Mean histo-score (H-score) as a measurement for DJ-1 expression in oral squamous cell carcinoma (OSCC) versus normal oral mucosa tissue samples. The bar graph shows the comparison of the mean H-scores for DJ-1 immunoreactivity in normal oral mucosa samples (N = 13) to OSCC samples (N = 143). ****p < 0.0001

Moreover, the mean H-score for DJ-1 immunoreactivity in normal oral mucosa tissues was 0.48, while the mean H-score for DJ-1 immunoreactivity in OSCC tissue samples was 1.42. Overall, there was a highly significant 2.98-fold increase of DJ-1 expression in OSCC in comparison to normal oral mucosa tissues (p > 0.0001) (Figure 2).

DJ-1 expression correlates with the histopathological grade of oral squamous cell carcinoma

**Figure 3:**
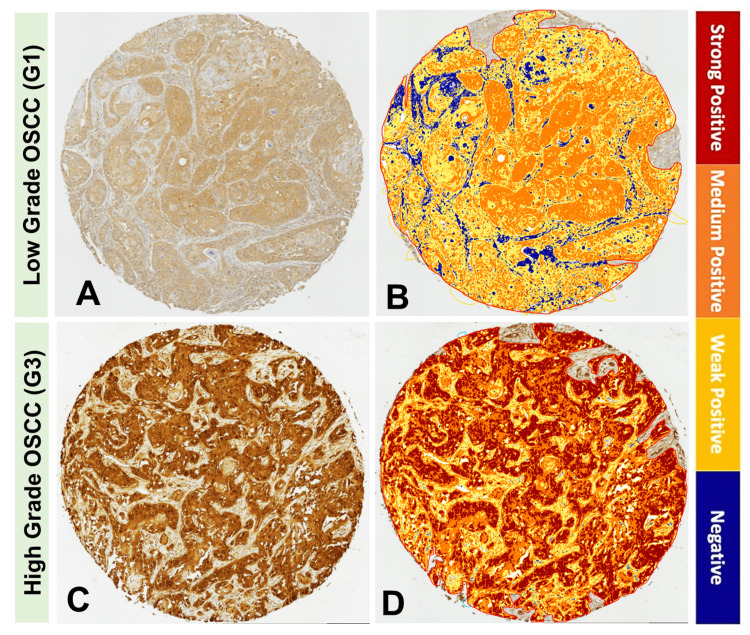
DJ-1 expression in oral squamous cell carcinoma (OSCC) tissue samples with low histopathological grades versus OSCC tissue samples with high histopathological grades. The upper panel shows a representative low histopathological grade (G1) OSCC tissue sample: (A) immunohistochemistry for DJ-1 immunoreactivity and (B) ImageScope computer-assisted analysis. The lower panel shows a representative high histopathological grade (G3) OSCC tissue sample: (C) immunohistochemistry for DJ-1 immunoreactivity and (D) ImageScope computer-assisted analysis.

Immunohistochemistry expression analysis showed that DJ-1 immunoreactivity was stronger in OSCC samples with high histopathological grades (G2 and G3) compared to OSCC samples with low histopathological grades (G1) (Figure 3).

**Figure 4:**
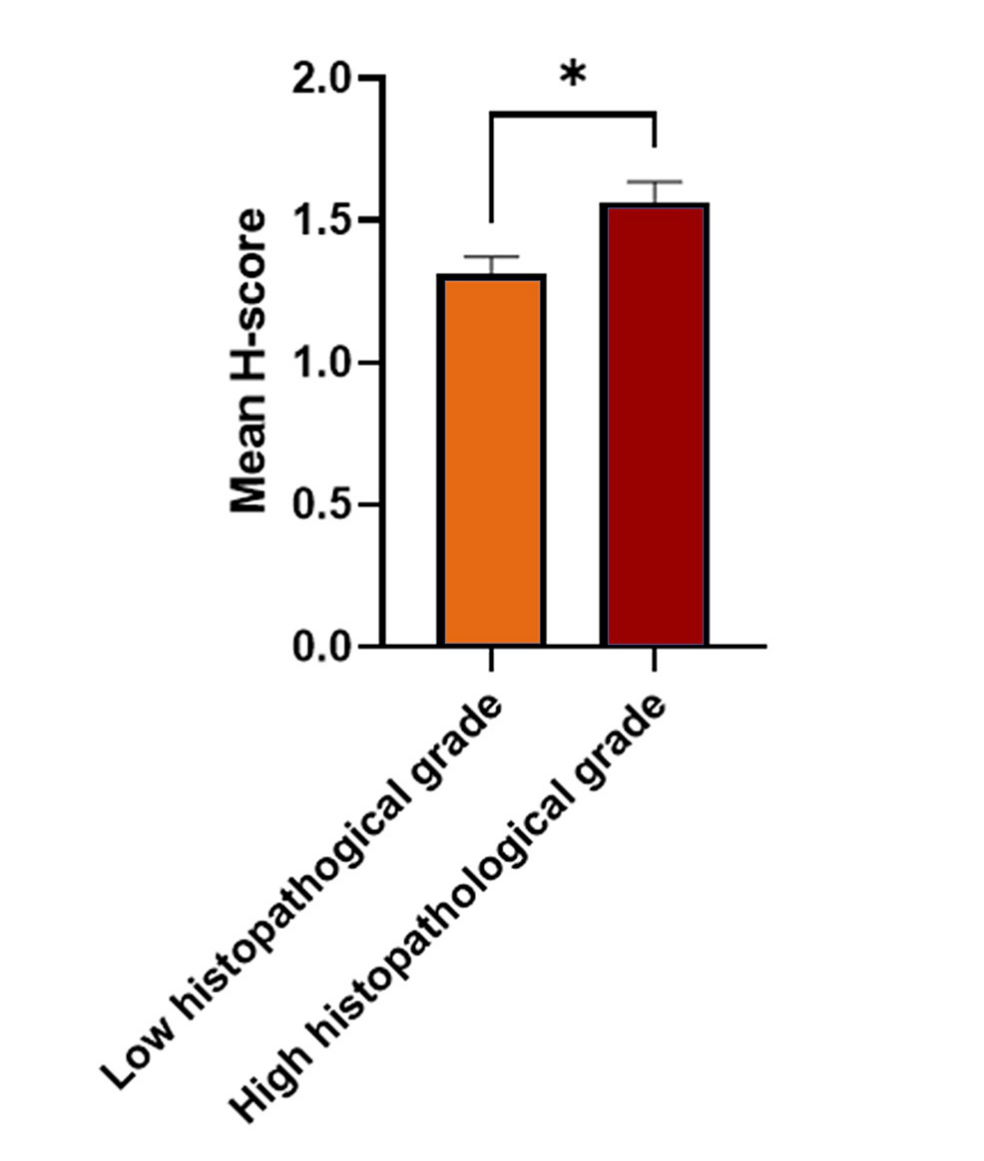
Mean histo-score (H-score) as a measurement for DJ-1 expression in oral squamous cell carcinoma (OSCC) tissue samples with low histopathological grades versus OSCC with high histopathological grades. The bar graph shows the comparison of the mean H-scores for DJ-1 immunoreactivity in OSCC with low histopathological grades (G1) (N = 80) to OSCC with high histopathological grades (G2 and G3) (N = 63). *p < 0.05

Computer-assisted quantitative analysis revealed a mean H-score of 1.56 in the 63 samples for high histopathological grade OSCC and a mean H-score of 1.31 in the 80 samples representing low histopathological grade OSCC. Overall, there was a 1.19-fold increase in DJ-1 expression in OSCC samples with high histopathological grades compared to OSCC samples with low histopathological grades (p = 0.01) (Figure 4).

## Discussion

The protein deglycase DJ-1 is an oncogene that is frequently upregulated and is associated with poor prognosis in several malignancies, such as glottic and supraglottic squamous cell carcinoma [10,13,14]. Importantly, the siRNA silencing of DJ-1 expression in cancer cell lines leads to reduced proliferation and enhanced apoptosis [17]. At the molecular level, DJ-1 binds and inhibits the tumor suppressor phosphatase and tensin homolog (PTEN) [18], which is a negative regulator of the phosphatidylinositol 3-kinase/protein kinase B (PI3K/Akt) signaling pathway involved in cancer cell survival, proliferation, and migration [14].

This study evaluated the utility of DJ-1 as a diagnostic marker for oral squamous cell carcinoma and found that DJ-1 expression was significantly greater in OSCC samples compared to normal oral mucosa tissues, regardless of the OSCC histopathological grade, consistent with the established role of DJ-1 as an oncogene [17,18].

Moreover, this study also documented a significant upregulation of DJ-1 expression in OSCC with higher histopathological grades compared to OSCC with low histopathological grades. This finding may bear significant utility as histopathological grade has been shown to affect survival rates in oral cancer patients [19]. A retrospective study in India has found that there was a high frequency of recurrence observed in patients with moderately differentiated oral squamous cell carcinoma. It also found that poorly differentiated tumors were associated with lower survival. The study ultimately concluded that the lower survival rate was due to the aggressive nature of poorly differentiated tumors in comparison to moderate and well-differentiated tumor cells and that histopathological grade had a significant correlation with the clinical characteristics of oral cancers [19]. This ultimately highlights the important role that tumor histopathological grade exerts as a prognostic indicator.

Taking into account that DJ-1 expression can differentiate between different grades of OSCC, this attribute may serve as a way to capture a more accurate picture of the biological behavior of a certain tumor given that the lack of differentiation within tumors of high histopathological grades is associated with highly aggressive behavior of malignant neoplasms [6,19]. As has been demonstrated with other cancers, these findings may ultimately help detect oral cancers at an early stage, forecast prognosis, and monitor tumor progression or response to therapy [20].

The limitations of this study are mainly related to the reliance on tissue microarrays, which consist of small cores of tissue samples that may not be representative of whole tumors, or reflective of the heterogeneity within a tumor, despite their advantages of profiling multiple samples for biomarker discovery in a rapid and uniform way [21,22]. Additionally, tissue microarray samples are typically archival in nature and lack real-time clinical data, such as survival and recurrence rates.

Therefore, follow-up studies are needed to monitor DJ-1 expression patterns in a large cohort of oral cancer patients in order to correlate these expression levels to relative survival rate and recurrence rate data and firmly establish the DJ-1 oncogene as a biomarker for oral cancer in the clinical setting and supplement the current methods of prognosis assessment.

## Conclusions

This study has shown that the DJ-1 oncogene is differentially expressed between normal oral mucosa and oral squamous cell carcinoma, as well as between the different histopathological grades within OSCC tissue samples. This study should add credence to the possible utility of the DJ-1 oncogene as an effective diagnostic tool in the assessment of head and neck cancers and in the determination of further treatment plans for patients suffering from oral cancer.
